# Stem cells in cancer therapy: opportunities and challenges

**DOI:** 10.18632/oncotarget.20798

**Published:** 2017-09-08

**Authors:** Cheng-Liang Zhang, Ting Huang, Bi-Li Wu, Wen-Xi He, Dong Liu

**Affiliations:** ^1^ Department of Pharmacy, Tongji Hospital, Tongji Medical College, Huazhong University of Science and Technology, Wuhan, Hubei Province, China; ^2^ Department of Oncology, Tongji Hospital, Tongji Medical College, Huazhong University of Science and Technology, Wuhan, Hubei Province, China

**Keywords:** stem cell, targeted cancer therapy, tumor-tropic property, cell carrier

## Abstract

Metastatic cancer cells generally cannot be eradicated using traditional surgical or chemoradiotherapeutic strategies, and disease recurrence is extremely common following treatment. On the other hand, therapies employing stem cells are showing increasing promise in the treatment of cancer. Stem cells can function as novel delivery platforms by homing to and targeting both primary and metastatic tumor foci. Stem cells engineered to stably express various cytotoxic agents decrease tumor volumes and extend survival in preclinical animal models. They have also been employed as virus and nanoparticle carriers to enhance primary therapeutic efficacies and relieve treatment side effects. Additionally, stem cells can be applied in regenerative medicine, immunotherapy, cancer stem cell-targeted therapy, and anticancer drug screening applications. However, while using stem cells to treat human cancers appears technically feasible, challenges such as treatment durability and tumorigenesis necessitate further study to improve therapeutic performance and applicability. This review focuses on recent progress toward stem cell-based cancer treatments, and summarizes treatment advantages, opportunities, and shortcomings, potentially helping to refine future trials and facilitate the translation from experimental to clinical studies.

## INTRODUCTION

Cancer is a leading cause of death in both developed and developing countries, and is an increasing medical burden worldwide, due to population growth and aging. Cancer is mainly treated using surgical resection, fractionated radiotherapy, and chemotherapy. However, treatment-related side effects, off-target effects, and drug resistance limit the efficacies of many therapeutic options. Furthermore, metastatic cancer cells usually cannot be eliminated by traditional therapies, and recurrence in these cases is extremely likely. Therefore, researchers are working to develop new, effective therapies with low or no toxicity in normal cells.

Stem cells have unique properties, such as migration toward cancer cells, secretion of bioactive factors, and immunosuppression, which promote tumor targeting and circumvent obstacles currently impeding gene therapy strategies. Preclinical stem cell-based strategies show great promise for use in targeted anti-cancer therapy applications. Nevertheless, there remain scientific concerns regarding the use of stem cell therapies, and further studies are needed to validate preclinical findings. This review summarizes recent anti-cancer stem cell therapy studies, and identifies advantages, opportunities, and potential challenges.

## STEM CELL DEFINITIONS AND SOURCES

As a unique population, stem cells are defined by their ability to: 1) self-renew indefinitely, 2) form single cell-derived clonal cell populations, and 3) differentiate into various cell types [1]. Self-renewal in resident stem cell pools plays key roles in tissue regeneration and homeostasis [2]. Stem cells can be broadly categorized as ‘embryonic’ (ESCs) or ‘somatic’ (SSCs). SSCs are also known as adult stem cells, which are generally multipotent and can differentiate into any cell type with a specific lineage, including neural stem cells (NSCs), mesenchymal stem cells (MSCs), hematopoietic stem cells (HSCs), endothelial progenitor cells (EPCs), and others. In at least some cases, cancer stem cells (CSCs) may drive tumorigenesis and disease progression [3].

### ESCs and iPSCs

As pluripotent cells, ESCs can differentiate into all cell types except those in the placenta [4], and are therefore used as gold standards in the evaluation of all pluripotent cells cultured *in vitro*. However, due to ethical considerations, applications for ESCs in scientific studies and human clinical trials are restricted. To this end, ESCs can be replaced by induced pluripotent stem cells (iPSCs) reprogrammed from adult somatic cells (e.g. skin fibroblasts) through enforced expression of pluripotency factors, because iPSC establishment does not require embryo destruction [5]. iPSCs are similar to ESCs, but lack immunogenic or ethical limitations, and so may be more clinically applicable than ESCs.

### NSCs

NSCs are typified by expression of nestin, Sox2, and other classic markers, together with expansion in culture media rich in epidermal and fibroblast growth factors [6]. NSCs can self-renew and differentiate into astrocytes, neurons, or oligodendrocytes, and have been widely employed to treat brain, breast [7], prostate [8], and lung [9] tumors.

### MSCs

MSCs are derived from bone marrow and can differentiate into mesodermal cells, including cartilage, bone, adipose tissue, stroma, muscle, connective tissue, and tendon. MSCs are easily isolated and propagated *in vitro* and, like NSCs, are applied widely in the treatment of different cancers.

### HSCs

HSCs, the most primitive of the blood lineage cells, are predominantly found in bone marrow, and produce mature blood cells through proliferation and differentiation of increasingly lineage-restricted progenitors. Transplantation of HSCs has been employed clinically for over four decades.

### EPCs

EPCs are the primary drivers of vascular regeneration [10]. Asahara, *et al.* suggest potential utility for EPCs in cancer therapy, following transfection or coupling with antitumor drugs or angiogenesis inhibitors [11]. However, recent advances have shifted the focus to EPC roles in disease pathogenesis and potential benefits as part of therapeutic interventions [10]. Reports on EPCs in cancer therapy are rare.

### CSCs

Based on cell surface markers, CSCs, a stem-like cancer cell subpopulation, are isolated from patient tissues and cell lines of different cancer types. CSCs express stemness genes, self-renew, differentiate into other non-stem cancer cells, and resist traditional cancer treatments [3]. CSCs likely initiate many cancer types. Traditional cancer therapies can kill non-stem cancer cells, but cannot eliminate CSCs. Tumors usually relapse when the remaining CSCs proliferate and differentiate. Therefore, targeting CSCs may solve clinical issues like drug resistance and recurrence [12].

## STEM CELL PROPERTIES

In addition to their self-renewal and differentiation capabilities, stem cells have immunosuppressive, antitumor, and migratory properties. Because stem cells express growth factors and cytokines that regulate host innate and cellular immune pathways [13, 14], they can be manipulated to both escape the host immune response and act as cellular delivery agents. Stem cells can also secret factors, such as CCL2/MCP-1, and physically interact with tumor cells, changing co-cultured tumor cell phenotypes and exerting intrinsic antitumor effects [15].

Importantly, many human stem cells have intrinsic tumor-tropic properties that originate from chemokine-cancer cell interactions. Stem cells first exhibited migratory capabilities in xenograft mouse models, manifested as tumor-homing abilities [16]. Possible stem cell migration mechanisms have been extensively studied. NSC migration to tumor foci is triggered by hypoxia, which activates expression of chemoattractants [6]. Directional HSC migration depends on the interaction between chemokine, CXCL12, and its receptor, CXCR4 [17]. A variety of MSC-expressed chemokine and growth factor receptors may participate in tumor homing [18]. The stromal cell-derived factor 1 (SDF1)/CXCR4 axis plays a major role in the migration of various stem cells [19–21]. To improve directed homing, stem cells have been engineered with higher levels of chemokine receptors, or target tissues have been manipulated to release more chemokines [22]. Park, et al. reported that CXCR4-overexpressing MSCs migrated toward glioma cells more effectively than control MSCs *in vitro* and in a xenografted mouse model of human glioma [20]. Controlled release of a chemokine from various biomaterials enhances recruitment of stem cells towards them. Schantz et al. achieved site-specific homing of MSCs toward a cellular polycaprolactone scaffold, which was constantly releasing SDF-1 with micro delivery device *in vivo* [23]. Thus, these two strategies can be combined to increase homing efficiency and improve treatment outcomes.

## STEM CELL MODIFICATIONS FOR CANCER THERAPY

Stem cells, most commonly NSCs and MSCs, can be modified via multiple mechanisms for potential use in cancer therapies. Common modifications include the therapeutic enzyme/prodrug system, and nanoparticle or oncolytic virus delivery at the tumor site.

### Enzyme/prodrug therapy

NSCs and MSCs can be engineered to express enzymes that convert non-toxic prodrugs into cytotoxic products. When modified stem cells are transplanted into tumor-bearing models, they localize to tumor tissues, where the exogenous enzyme converts the prodrug into a cytotoxic molecule, ultimately damaging the tumor cells. As a result, the amount, timing, and location of drug release can be precisely controlled. Enzyme/prodrug therapy is also called suicide gene therapy, and was the first engineered NSC therapeutic application and the first to enter clinical trials [16, 24].

Cytosine deaminase (CD) is a major enzyme currently used in enzyme/prodrug therapy. CD converts the prodrug, 5-fluorocytosine (5-FC), into the toxic variant, 5-fluorouracil. Aboody, *et al.* reported that the combination of CD-bearing mouse NSCs and 5-FC inhibited glioblastoma (GBM) cell growth [16]. Injecting CD-expressing MSCs into the brain with 5-FC also suppressed tumor growth [25]. In one of the most commonly used cytotoxic therapies, human HB1.F3 cells are engineered to express CD (HB1.F3.CD) [26]. With outstanding efficacy and safety, HB1.F3.CD/5-FC therapy was recently applied in the first human clinical trial (ClinicalTrials.gov identifier: NCT01172964), in which HB1.F3.CD cells were injected into the cavity wall following GBM resection, and patients received oral 5-FC [24]. This study was completed, and results have not yet been released. Another trial (ClinicalTrials.gov identifier: NCT02015819) using modified NSCs to treat glioma will be completed in October 2018.

Herpes simplex virus-thymidine kinase (HSV-TK) has also been utilized in suicide gene therapy [27]. HSV-TK phosphorylates the prodrug, monophosphorylate ganciclovir (GCV), to produce cytotoxic triphosphate ganciclovir (GCV-TP). GCV-TP integrates into the DNA of nearby cells during division, leading to cell death via DNA polymerase inhibition. Li, *et al.* reported that C6 gliomas in rats were effectively treated by intratumoral HSV-TK-transduced NSC (NSC-TK) injection followed by intraperitoneal GCV injection daily for 10 days (two 15 mg/kg doses/day). Six of nine rats survived 100 days post-injection, without any signs of tumor [28]. Another study showed that NSCs-TK injected into the brain migrated to the contralateral hemisphere, co-localized with U87 cells, and conferred long-term survival on GCV-treated mice [29].

### Secreted agents

Stem cells can function as *in situ* drug factories, secreting antitumor agents for an extended period of time, and overcoming various cancer therapy limitations, such as high systematic toxicity and short drug half-life. TNF-α-related apoptosis-inducing ligand (TRAIL) is one of the most widely used, secreted therapeutic agents, and induces tumor cell apoptosis [30]. However, its short half-life reduces its therapeutic effectiveness *in vivo* [31]. This could be mitigated by encapsulating TRAIL-expressing stem cells in a synthetic extracellular matrix (sECM) that is introduced into the GBM resection cavity after surgical debulking [32]. The encapsulated cells could continually release therapeutic molecules at resection margins. This approach delays malignant and invasive brain tumor regrowth and increases survival in mice.

Stem cells can also be modified to selectively deliver growth inhibitory proteins (e.g. IFN-β), rendering the microenvironment inhospitable to tumor growth. Ling, *et al.* studied the migration of IFN-β-expressing MSCs and their engraftment into primary breast tumor sites, and found that tumor cell growth was suppressed, and hepatic and pulmonary metastases were alleviated [33]. MSCs secreted IFN-β at high levels in the tumor microenvironment but not in the circulation. This study also suggested that *in situ* IFN-β expression in MSCs suppressed or abrogated cancer cell growth by inactivating signal transducer activator transcription factor 3 (Stat3).

### Viral therapy

Oncolytic viruses (OVs), unlike traditional attenuated viruses, conditionally replicate in tumor cells. OVs have increased spread in the body and hide from the immune system. OV-transduced NSCs are still able to home to tumor cells, and NSC-delivered OVs showed better antitumor effects than the viruses alone against GBMs *in vivo* [34]. Similarly, after radiotherapy and temozolomide treatment, NSC-delivered OVs increased survival in glioma bearing mice [35]. Early clinical trials for antiglioma gene therapies based on adenovirus vectors reported sufficient tolerabilities without serious adverse events [36].

Virus delivery by MSCs is also a promising approach for targeted cancer therapy. Ong, *et al.* demonstrated that the potent oncolytic activity of attenuated measles virus combined with the unique immunoprivileged and tumor-tropic properties of MSCs could combat hepatocellular carcinoma [37]. Systemically delivered measles virus-infected MSCs homed to tumors implanted orthotopically in the liver and transferred MV infectivity to cancer cells via heterofusion, inhibiting tumor growth. Duebgen, *et al.* showed that MSC-mediated delivery of oncolytic herpes simplex virus (oHSV) in a GBM resection mouse model enhanced the virus’ antitumor effects [38]. In this approach, oHSV produced by MSC dynamically infected GBM cells, killing tumor cells *in vitro* and *in vivo*. Combining oHSV with TRAIL may also effectively avoid resistance in tumors. oHSV/TRAIL-loaded MSCs effectively induced tumor cell apoptosis and extended median survival time in mice bearing oHSV- and TRAIL-resistant GBMs [38].

### Nanoparticle carriers

Delivery systems based on nanoparticle carriers (NPs) often contain high-concentration insoluble chemotherapeutic reagents, and protect them from degradation in a harsh biological environment. Failure to target micrometastatic lesions, inefficient dissemination in solid tumors, and other limitations can be overcome by using stem cells as NP delivery agents [39, 40]. Stem cells can also reduce unrestricted uptake of nanoparticles by mononuclear cells and protect therapeutic agents from host immunosurveillance [39]. Roger, et al. found that MSCs efficiently internalized NPs and could act as NP delivery vehicles in brain tumors [41]. MSC cell membranes can be loaded with doxorubicin-containing porous silica nanorattles for tumor-tropic therapy [42]. This approach increased and extended intratumoral drug distribution and promoted tumor cell apoptosis more than free drug or drug delivery systems using silica nanorattles alone. Thus, stem cell-mediated NP-based drug delivery shows great promise in cancer treatments, and warrants further investigation.

## OTHER APPLICATIONS OF STEM CELL IN CANCER THERAPY

### Regenerative medicine

Given their self-renewal and differentiation capabilities, stem cells can be used to repair human tissues after chemotherapy. Transplanting HSCs has been widely clinically employed to facilitate lifelong hematological recovery after treatment of malignancies with high-dose radiotherapy or chemotherapy. This treatment aims to reconstitute bone marrow under marrow failure conditions (e.g. aplastic anemia) and to treat blood cell genetic diseases, and works by supplying stem cells that differentiate into a desired type of blood cell. Transplantation and successful engraftment of only one HSC can reconstitute hematopoiesis in recipients [43, 44].

Healthy iPSCs derived from patient tissues can theoretically be employed to regenerate tumor- or treatment-injured tissues. In regenerative medicine, various tissues can be produced using iPSCs. iPSC therapy may be useful in repairing or replacing cancer patient iPSCs damaged by chemotherapy, radiotherapy, or surgical treatment. However, regenerative therapy mediated by human iPSCs requires robust *in vivo* engraftment of iPSC-derived tissues. Currently, only a few types of human iPSC-derived cells (e.g. hepatocytes) have been successfully engrafted in animal models [45, 46].

### Immunotherapy

An immune-mediated antitumor effect following allogeneic HSC transplantation might be sufficient to cure some hematological malignancies [47–49]. Introducing genes encoding chimeric antigen receptors (CARs) or T-cell receptors (TCRs) directed against tumor-associated antigens makes HSCs attractive for use in cancer immunotherapy [50, 51].

Patient-specific iPSCs could also potentially benefit immunotherapy approaches [52, 53]. The pre-rearranged TCR gene is retained in T lymphocyte-derived human iPSCs, which can be further induced to differentiate into functionally active T cells [54–56]. Functional, tumor antigen-specific T lymphocytes can be produced *in vitro* by reprogramming selected T cells into iPSCs which then differentiate back into T lymphocytes for infusion into patients. However, the safety of T cell-derived human iPSCs must be further validated.

### Targeting CSCs

CSCs are multipotent, can self-renew, and have high proliferative capacities, contributing to rapid activation of tumor invasion and metastasis. Therefore, targeting CSCs is vital to ensuring high therapeutic efficacies and preventing tumor recurrence [3]. Since CSCs can attract normal stem cells, normal stem cells can be potentially used to target CSCs in cancer therapy. Interactions between normal stem cells and CSCs suppress tumor proliferation, angiogenesis, and metastasis, and reduce inflammation and apoptosis. Bryukhovetskiy, et al. assessed the potential of NSCs and HSCs in anti-glioblastoma therapy [57], and concluded that HSCs may be ideal for developing technologies aimed at controlling glioblastoma CSC activity, as HSCs are less prone to neoplastic transformation in neural tumors than NSCs. Similarly, engineered HSCs may facilitate the generation of cell systems that can trigger targeted CSC apoptosis [58].

### Anticancer drug screening

In addition to treating cancers directly, iPSCs can be used to screen new anticancer drugs. Differentiating patient cancer tissue-derived iPSCs generates cell types that may be more biologically related to human tumors than currently available drug screening methods, such as traditional cancer cell lines, mouse xenograft models, and mouse tumors. Additionally, hepatotoxicity prevents many potential antitumor drugs from being clinically applied, and can be screened for using hepatocytes produced from human iPSCs with various genetic backgrounds [59]. The applications of stem cells in cancer therapy are listed in Table 1.

**Table 1:** Applications of stem cells in cancer therapy

Strategies	Cancer types	Stem cell applications	References
Stem cell modifications			
Enzyme/prodrug therapy	Glioma	NSCs (retroviral transduction with CD)	[16, 26]
		NSCs (baculoviral transduction with HSV-TK)	[29]
		MSCs (lentiviral and retroviral transduction with S-TRAIL and HSV-TK)	[27]
		MSCs (retroviral transduction with CD )	[25]
	Colon adenocarcinoma	NSCs (adenovirus transduction with a rabbit CE)	[26]
	Metastatic lung cancer and primary lung cancer	NSCs (engineered to express CE)	[9]
Secreted agents	Glioma	NSCs (retrovirus transduction with IL-4 )	[26]
		NSCs (adenovirus transduction with TRAIL)	[26]
		NSCs (encapsulated in sECM after being engineered to express S-TRAIL)	[32]
	Breast cancer brain metastases	NSCs (lentivirus transduction with anti-HER2Ab)	[7]
	Breast cancer	MSCs (engineered to over express IFN-beta)	[33]
Viral therapy	Glioma	NSCs (infected with CRAd-S-pk7)	[35, 67]
		MSCs (loaded with oHSV )	[38]
	Hepatocellular carcinoma	MSCs (infected with measles virus)	[37]
Nanoparticle carriers	Solid tumor	NSCs (loaded with gold nanorods)	[40]
	Glioma	MSCs (loaded with poly-lactic acid nanoparticles and lipid nanocapsules)	[41]
		MSCs (loaded with nanoparticles)	[42]
Regenerative medicine	Hematologic malignancies	HSCs (allogeneic transplantation)	[44]
	Liver disease	iPSCs (engraftment of patient-specific iPSCs)	[45, 46]
Immunotherapy	Solid tumor	HSCs (induction of graft vs. tumor effect )	[48]
	Lymphomas	HSCs (allogeneic transplantation)	[49]
		iPSCs (generate T cells)	[53]
	Melanoma	HSCs (genetically engineered HSCs to generate antigen-specific CD8 T cells)	[51]
Targeting CSCs	Glioma	HSCs (modifying the proteome profile of HSCs )	[57]
Anticancer drug screening	/	cancer tissue-derived iPSCs (provide cellular targets)	[59].

## FACTORS INFLUENCING STEM CELL THERAPIES

### Stem cell type

While stem cells share similar properties, their therapeutic effects may differ. Ahmed, *et al.* first compared NSCs with MSCs as carriers for an oncolytic adenovirus in a glioma model. Both stem cell types supported intracellular adenoviral replication, but a log more virus was released from NSCs than from MSCs (*p* < 0.001). Additionally, only intracranial administration of virus-loaded NSCs prolonged survival in an animal model of orthotopic glioma (median survival for MSCs: 44 days vs. that for NSCs: 68.5 days, *p* < 0.002) [60]. NSCs exhibited superior therapeutic efficacy in intracranial tumors compared to MSCs, despite comparable migration capacities, suggesting that carrier trafficking efficacy may be closely linked to the level of relatedness between carrier origin cells and malignant cells [60].

In anti-cancer therapy, the choice of stem cell type depends on cell-specific characteristics and therapeutic requirements. To treat hematologic and non-hematologic malignancies, autologous HSC transplantation is frequently used to rescue hematopoiesis after high-dose chemotherapy. Upon congenital and acquired marrow failures, this method is also widely used to continuously meet mature blood cell replenishment requirements [61]. iPSCs are also better than other stem cells for assessing candidate antitumor drug toxicities [62].

### Route of transplantation

The route of stem cell delivery plays a critical role in anti-tumor therapy [60]. An appropriate method must consider target pathology, therapeutic objectives, and patient risk-benefit profile. In murine models of GBM, efficient NSC delivery is achieved via contralateral injection into the tumor site [63]. However, intracranial injections are invasive and not ideal for repeated operations. NSCs delivered intranasally can still efficiently migrate to tumor tissues [64], allowing repeated administration [65]. This approach can also avoid intravascular delivery-related complications, such as pulmonary embolism, obstruction by the blood brain barrier, and infarctions [64].

Compared with cell suspension injections, semisolid substrates may augment transplantation efficiencies by providing mechanical support and relieving metabolic stress. Currently, poor survival of NSC grafts can be tentatively counteracted by transplantation of stem cells utilizing biocompatible devices. Hansen, *et al.* reported a three-dimensional extracellular matrix-based substrate (3DECM), purified from engineered skin cultures, that could provide an efficient clinical administration route for cell grafts. 3DCEM enabled *in vitro* expansion of embedded NSCs, retaining their uncommitted differentiation [66].

### Cell number and transplantation timing

Treatment outcomes are affected by transplanted cell numbers and transplantation timing. Transplantation of an insufficient number of HSCs in patients with oncohematological diseases results in inefficient hematopoietic component replacement, and diseases easily relapse [61]. However, a too-large number of transplanted cells may increase the risk of teratoma formation or ectopic engraftment. Thus, the number of cells for effective treatment should be optimized.

Stem cell therapy efficacy depends on administration timing. For example, NSCs should be given before ionizing radiation (XRT) and temozolomide (TMZ). Alex, *et al.* reported that loaded NSCs given to GBM43 xenografted animals prior to XRT-TMZ treatment increased median survival by 9 days over that of animals receiving a reverse schedule (*p* < 0.05) [35]. Additionally, 33% of mice receiving loaded NSCs prior to TMZ-XRT lived ≥ 70 days, compared to only 9% of mice receiving the reverse regimen. Furthermore, loaded NSC administration before XRT-TMZ treatment promoted mouse brain tumor cell apoptosis.

For oncolytic virotherapy, carrier cells must first accumulate in tumor beds. Then, viral progeny are released to allow targeted delivery of the functioning virus. Thaci, *et al.* found that maximum viral progeny were released from NSCs seven days after loading *in vitro*; ideally, carrier cells should reach tumor sites prior to this time [67]. Most NSCs migrated to tumor sites within 24–48 hours after implantation [68]. Thus, oncolytic viruses delivered via NSCs should have replication cycles appropriate for NSC tumor-homing abilities.

## CHALLENGES TO STEM CELL THERAPY

### Treatment durability

Tumors commonly relapse regardless of strong initial therapeutic effects. Like most chemotherapies, stem cell therapy using a single agent generally cannot eliminate tumors. Therefore, an optimum drug combination should be rationally selected [6]. Many combination therapies have been tested to improve treatment durability. For example, IFN-β immunotherapy combined with chemotherapy using a prodrug/suicide gene system has shown synergistic therapeutic effects against human colorectal cancer [69]. Irradiating tumor cells can induce production of factors that stimulate MSC invasion through integral basement membranes, increasing the number of MSCs in tumors [70]. Combining stem cell-based oncolytic virotherapy with chemoradiotherapy can minimize residual disease volumes and sensitize glioma cells to CRAd-S-pk7 (OV CRAd-Survivin-pk7) during radiotherapy [35]. Kim, *et al.* [71] found that TMZ sensitized glioma cells to TRAIL-induced apoptosis by modulating the apoptotic machinery, and enhanced MSC-TRAIL gene therapy antitumor effects. Epidermal growth factor receptor (EGFR), which is mutated and overexpressed in various tumors, is associated with poor prognosis and shortened survival [72]. TRAIL combined with stem cell-delivered immunoconjugates of EGFR-specific nanobodies enhanced treatment outcomes [73].

### Potential tumorigenesis concerns

Normal stem cells share some characteristics with CSCs, including self-renewal, differentiation, and epithelial-to-mesenchymal transition capacities. Stem cell therapy may increase cancer risk, as evidence by tumor formation four years after fetal neural stem cell transplantation for ataxia-telangiectasia [74]. Thus, prevention of tumor formation by transplanted stem cells requires additional study [63]. However, whether stem cells promote the growth of certain tumors or form tumors themselves is uncertain. Karnoub, *et al.* demonstrated that bone-marrow-derived MSCs mixed with otherwise weakly metastatic human breast carcinoma cells increased the cancer cells’ metastatic potentials, allowing for tumor formation in subcutaneous xenografts [75]. The breast cancer cells promoted MSC secretion of chemokine CCL5, which acted in a paracrine fashion to increase cancer cell motility, invasion, and metastasis. Increased breast cancer cell metastatic capability was reversible and dependent on CCL5 signaling through the chemokine receptor, CCR5. Therefore, MSCs in the tumor microenvironment facilitated metastasis by reversibly changing cancer cell phenotypes.

Rosland, *et al.* [76] showed that spontaneous malignant transformation occurred in 45.8% (11/24) of bone marrow-derived MSC long-term (5–106 weeks) cultures, indicating spontaneous malignant transformation. *In vitro* cell culture conditions may initiate stress-induced genomic instability, promoting the malignant phenotype. Mutation tendency has also been related to oxygen tension [77] and matrix elasticity [78]. Therefore, optimization of *in vitro* culture conditions is important for MSC expansion for clinical use. However, other groups present contradictory findings regarding MSC transformation tendencies. Bernardo, *et al.* reported that MSC remain stable and do not transform in long-term cultures [79]. Thus, stem cell fates may be largely dependent on culture environments, and implanted stem cells may contribute to the growth of certain tumors or produce tumors themselves.

Multipotent NSCs, MSCs, and HSCs appear safer for clinical use than ESCs and iPSCs. Most studies focus on pluripotent stem cells that may be highly tumorigenic. There are six strategies to eliminate any possibility of neoplastic transformation [80]. First, undifferentiated pluripotent stem cells, which are potentially tumorigenic, can be excluded from clinical preparations using antibodies that target specific surface-displayed biomarkers. Stem cell differentiation downregulates display of these biomarkers. Monoclonal antibodies may facilitate fluorescence activated cell sorting or magnetic activated cell sorting of undifferentiated, pluripotent stem cells modified with fluorochromes or superparamagnetic chelates, respectively. Second, directed differentiation of iPSCs includes monitoring the expression of differentiation lineage-specific genes. Successfully differentiated cells can be identified and sorted using recombinant reporter proteins. GFP and similar proteins work well as reporters of undifferentiated vs. differentiated cells. Undifferentiated pluripotent stem cells transformed to express GFP emit telltale fluorescence upon illumination with specific wavelengths as long as they remain undifferentiated. This facilitates their sorting out or eradication through laser ablation. Third, undifferentiated cells can be killed using toxic antibodies or antibody-guided toxins. For example, monoclonal antibodies against claudin-6, a biomarker for undifferentiated pluripotent ESCs and iPSCs, can guide toxins to these stem cells for selective, targeted killing [81]. Fourth, undifferentiated stem cells can be eradicated using cytotoxic agents, which can be applied to selectively kill pluripotent stem cells that could develop into tumors. PluriSIn#1 inhibits stearoyl-CoA desaturase-1, an enzyme involved in monounsaturated fatty acid metabolism, and induces apoptosis in treated cells [82]. PluriSIn#1 treatment selectively eliminates undifferentiated iPSCs and ESCs [83]. Fifth, potentially tumorigenic stem cells can be sensitized to prodrugs through transformation using suicide genes. The enzyme/prodrug cancer therapy strategy can also be adapted to kill undifferentiated stem cells. For example, hESCs engineered to express the HSV-TK gene were killed following GCV treatment, whereas non-transfected hESCs were unaffected [84]. Finally, differentiated refractive stem cells can be eliminated through self-induced transgenic expression of recombinant human DNases. To this end, and to improve treatment safeties and efficacies, a toxic reagent-independent feedback loop was developed to select for differentiated stem cells [85]. iPSCs were directed to differentiate into endothelial or myocardial lineages, and were then transfected with human recombinant DNASE1, DNASE1L3, DNASE2, and DFFB, guided by antiSSEA-4 and anti-TRA-1-60 synthetic antibodies. Transgenes were delivered only to pluripotent, differentiation-refractive stem cells. Thus, iPSCs that maintained their pluripotency and specific cell surface display profiles, and continued proliferating instead of differentiating, expressed the human recombinant DNases. Genomic DNA was degraded in these potentially tumorigenic stem cells, ultimately killing the cells. These six strategies could safeguard against tumor transformation in stem cell population.

## CONCLUSIONS

Stem cell technologies may open new doors for cancer therapy. Stem cells migrate to solid tumors and micrometastatic lesions, facilitating site-specific anti-tumor agent delivery. Stem cells can be engineered to stably express a variety of antitumor agents, overcoming the short half-lives of conventional chemotherapeutic agents. However, conquering stem cell therapy limitations will require additional research to better illuminate relationships between normal and cancer stem cells. A better understanding of fundamental stem cell mechanisms will improve stem cell-based regenerative medicine and anti-cancer strategies, and is imperative for more widespread clinical utilization of stem cell-based therapies.

## Abbreviations

ESCs: embryonic stem cells
SSCs: somatic stem cells
HSCs: hematopoietic stem cells
NSCs: neural stem cells
MSCs: mesenchymal stem cells
iPSCs: induced pluripotent stem cells
EPCs: embyronic progenitor and stem cells
CSCs: cancer stem cells
SDF1: stromal cell-derived factor 1
CXCR4: CXC-chemokine receptor 4
CXCL: CXC-chemokine ligand
5-FC: 5-fluorocytosine
GBMs: glioblastomas
CD: deaminase
CE: carboxylesterase
anti-HER2Ab: anti epidermal growth factor receptor 2 antibody
HSV-TK: herpes simplex virus-thymidine kinase
GCV: ganciclovir
GCV-TP: triphosphate ganciclovir
TRAIL: TNF α-related apoptosis-inducing ligand
S-TRAIL: secretable TNF α-related apoptosis-inducing ligand
sECM: synthetic extracellular matrix sECM
OV: oncolytic viral
NP: Nanoparticle
IFN-β: interferon-β
Stat3: signal transducer activator transcription factor 3
oHSV: oncolytic herpes simplex virus
CARs: chimeric antigen receptors
TCR: T-cell receptors
3DECM: 3-dimensional extracellular matrix-based substrate
XRT: ionizing radiation
TMZ: temozolomide
CRAd-S-pk7: OV CRAd-Survivin-pk7
EGFR: Epidermal growth factor receptor EGFR
